# Divergence time estimation of Galliformes based on the best gene shopping scheme of ultraconserved elements

**DOI:** 10.1186/s12862-021-01935-1

**Published:** 2021-11-22

**Authors:** De Chen, Peter A. Hosner, Donna L. Dittmann, John P. O’Neill, Sharon M. Birks, Edward L. Braun, Rebecca T. Kimball

**Affiliations:** 1 MOE Key Laboratory for Biodiversity Science and Ecological Engineering, College of Life Sciences, Beijing Normal University, Beijing, China; 2 Department of Biology, University of Florida, Gainesville, FL USA; 3 Natural History Museum of Denmark and Center for Global Mountain Biodiversity, University of Copenhagen, Copenhagen, Denmark; 4 Museum of Natural Science, Louisiana State University, Baton Rouge, LA USA; 5 Burke Museum of Natural History and Culture, University of Washington, Seattle, WA USA

**Keywords:** Data heterogeneity, Fossil calibration, Galliformes, Molecular dating, PartitionFinder, Phylogenomics, Ultraconserved elements

## Abstract

**Background:**

Divergence time estimation is fundamental to understanding many aspects of the evolution of organisms, such as character evolution, diversification, and biogeography. With the development of sequence technology, improved analytical methods, and knowledge of fossils for calibration, it is possible to obtain robust molecular dating results. However, while phylogenomic datasets show great promise in phylogenetic estimation, the best ways to leverage the large amounts of data for divergence time estimation has not been well explored. A potential solution is to focus on a subset of data for divergence time estimation, which can significantly reduce the computational burdens and avoid problems with data heterogeneity that may bias results.

**Results:**

In this study, we obtained thousands of ultraconserved elements (UCEs) from 130 extant galliform taxa, including representatives of all genera, to determine the divergence times throughout galliform history. We tested the effects of different “gene shopping” schemes on divergence time estimation using a carefully, and previously validated, set of fossils. Our results found commonly used clock-like schemes may not be suitable for UCE dating (or other data types) where some loci have little information. We suggest use of partitioning (e.g., PartitionFinder) and selection of tree-like partitions may be good strategies to select a subset of data for divergence time estimation from UCEs. Our galliform time tree is largely consistent with other molecular clock studies of mitochondrial and nuclear loci. With our increased taxon sampling, a well-resolved topology, carefully vetted fossil calibrations, and suitable molecular dating methods, we obtained a high quality galliform time tree.

**Conclusions:**

We provide a robust galliform backbone time tree that can be combined with more fossil records to further facilitate our understanding of the evolution of Galliformes and can be used as a resource for comparative and biogeographic studies in this group.

**Supplementary Information:**

The online version contains supplementary material available at 10.1186/s12862-021-01935-1.

## Background

Divergence time estimation is fundamental to understanding many aspects of the evolution of organisms, such as character evolution, diversification, and biogeography [1, 2]. Due to the development of high through-put data generation, faster and better programs for data analyses, and improved fossil calibrations, several questions that used to be controversial have now been generally resolved. For example, both Neornithes birds and placental mammals are now considered to have diversified prior to rather than after the Cretaceous to Paleogene (K-Pg) mass extinction [3–6], even when different datasets and molecular clock methods are used [7].

Robust molecular dating depends on several factors, with the most critical being the use of suitable fossil calibration points, a broadly sampled and well-resolved phylogenetic topology, and adequate application of molecular clock methodologies [8]. Although the fossil record is limited, making selection of suitable fossils challenging, guidelines for selection and placement of available fossils have been developed that can lead to improved divergence estimates [9]. Additionally, use of fossils whose taxonomic placement is agreed upon [10], and assessment of whether certain fossils have an undue impact on date estimations [11], are improving our use of fossil calibrations.

Well-supported phylogenies, based on loci throughout the genome, are now available for many groups. Whole and reduced representation genome sequencing methods (e.g., transcriptome sequencing, sequence capture) provide hundreds to thousands of loci throughout the genome and have significantly improved phylogenetic estimation [12, 13]. Particularly, sequence capture of ultraconserved elements (UCEs) has shown great promise in resolving problematic short internodes in phylogenetic estimation, including those that had been controversial [14–16]. UCEs have a conserved core region [17] with flanks on each side that include more variable sites. The conserved regions facilitate sequence capture and the flanking regions provide phylogenetic signal [18]. UCE data have been used across a variety of vertebrate and invertebrate lineages in recent years [19–22]. UCE data have also been used to generate trees that include almost all recognized species for some major groups, either by combining UCEs with mitochondrial data and small numbers of nuclear loci [23] or using novel UCE data alone [24]. In addition, UCE sequence capture is especially promising for the generation of complete phylogenies since it can be used with degraded museum tissues [25], allowing previously unsampled taxa to be included in modern phylogenies [26, 27]. Thus, it is now not only possible to obtain well-supported phylogenies, but also ones that have broad representation within clades.

However, while UCEs and other phylogenomic datasets show great promise in phylogenetic estimation, the best ways to leverage the large amounts of data for divergence time estimation has not been well explored. These datasets are large, which can place computational burdens on the programs used to estimate divergence time (particularly for some methods, e.g., BEAST [28]). More importantly, however, these datasets (whether UCEs or other markers extracted from whole genomes) contain a mixture of heterogenous loci that evolve at different rates, may be under varying levels of selection (sometimes clade-specific), and differ in best-fitting models of evolution. This type of heterogeneity can mislead divergence time estimation [29], suggesting that it may be best to focus on a subset of data for divergence time estimation. However, a major question is which loci or partition (s) should be used for analyses. Should we identify the most “clock-like” loci or partition [3, 21], select the most “tree-like” loci or partition (that one that shows the least topological conflict with a focal species tree) [30], or use the largest partition [31]? While different approaches have been used to select subsets of data, exploration of how selection of the appropriate data subset affects divergence time estimation is rarely considered. In this study, we tested the effects of a series of “gene shopping” [32] schemes (selection of data subsets) on divergence time estimation from UCEs from the avian order Galliformes.

The Galliformes (gamebirds or landfowl) includes some of the best-studied avian species (e.g., chicken, turkey, and Japanese quail). Recent taxonomies [33], and molecular studies [26, 34] have consistently identified five families: Megapodiidae (mound builders), Cracidae (guans and chachalacas), Numididae (guineafowl), Odontophoridae (New World quail), and the largest family, Phasianidae (pheasants, partridges, and Old World quail). However, phylogenetic relationships using traditional molecular markers have shown conflict or low resolution within these families across studies [34, 35]. More recently, UCEs have resolved most generic relationships within two families, Phasianidae and Cracidae, with confidence [11, 26, 36]. However, within Megapodiidae, Numididae, and Odontophoridae there has been little sampling of UCEs. Instead, their relationships are primarily known from limited nuclear introns and mitochondrial genes, which show inconsistency [37, 38]. Thus, it is important to improve sampling of UCEs throughout this order to provide a good estimate of relationships across key nodes.

In this study, we had three main goals. First, we wanted to estimate a robust phylogeny sampled broadly through the entire order by obtaining UCEs from at least one representative of each genus. Second, using this phylogeny, we wanted to test the effects of different “gene shopping” schemes on divergence time estimation using a carefully, and previously validated, set of fossils [10, 11, 31]. Finally, using the most appropriate scheme, we wanted to determine divergence times throughout galliform history.

## Results

### Phylogenetic relationships within Galliformes

We obtained UCEs from four outgroups and 130 galliform species (Additional file 4: Table S1), including representatives of all genera, to generate a dataset including 135 taxa. The lengths of the 5026 aligned UCE loci ranged from 137 to 2322 bp (mean = 425 bp). The 75% complete matrix (greater than 75% of taxa present for each UCE locus) contained 3574 UCE loci that ranged from 216 to 1467 bp in length (mean = 443 bp). The total length of the 75% complete matrix was 1,584,884 bp, with 225,065 informative sites.

**Fig. 1:**
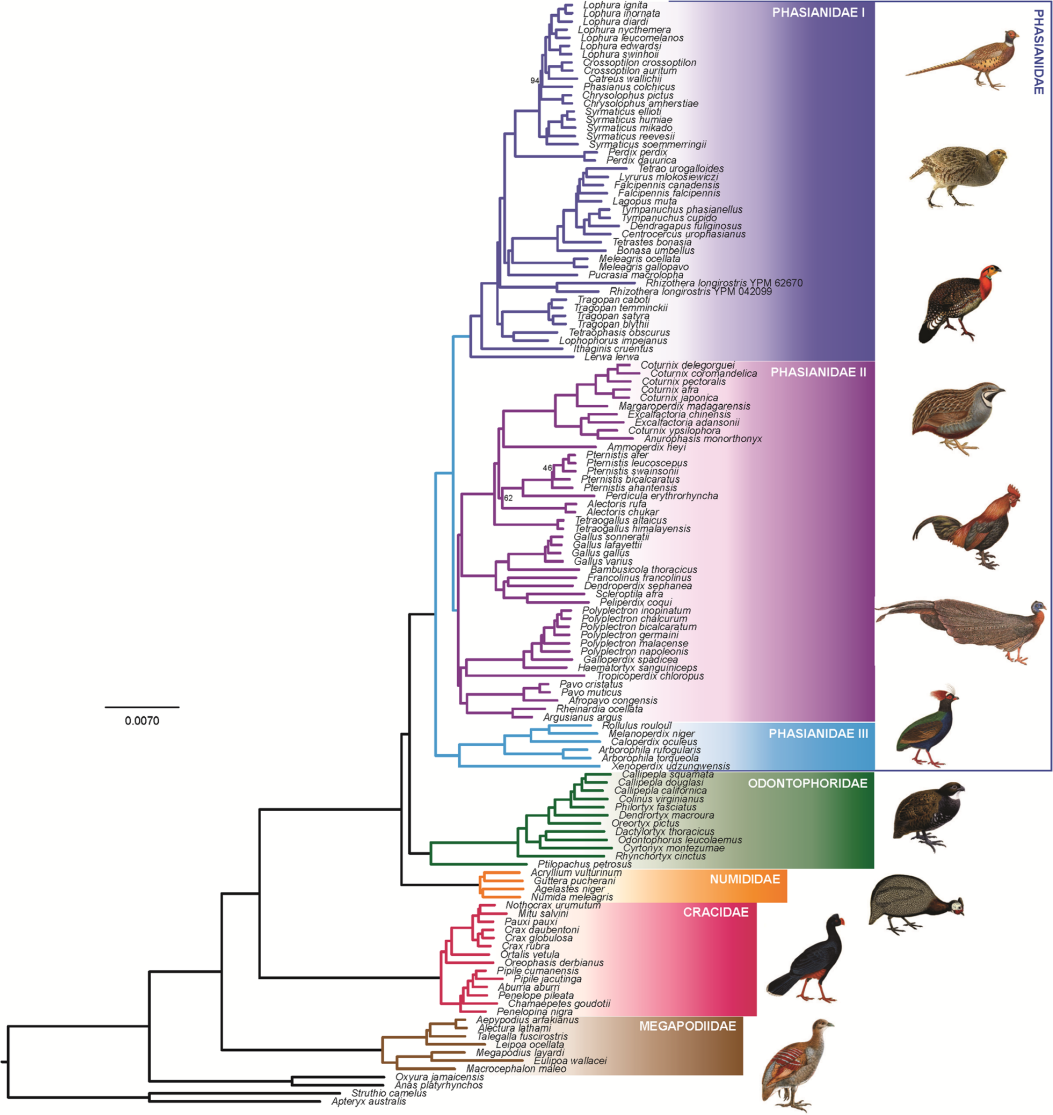
ML estimate of phylogeny for the 135-taxon Galliformes dataset using 100 bootstrap replicates. The 75% complete matrix was used for this analysis. Family names are indicated using colors and the boxes placed to the right of the tree. Phasianidae, the most species-rich clade in Galliformes, is broken into three subgroups: (I) the “erectile clade” (gallopheasants, true partridges, grouse, turkeys, tragopans, and allies); (II) the “non-erectile clade” (Old world quail, junglefowl, francolins, snowcocks, peafowl, peacock-pheasants, argus pheasants, and allies); and (III) Arborophilinae (Hill partridges and allies). We have used a blue box to the left of the tree to indicate that all three of these groups are members of Phasianidae. All but three branches in this tree have 100% bootstrap support (the three cases with < 100% support are indicated by support values adjacent to the relevant nodes

We estimated a ML phylogenetic tree from the 75% complete matrix by concatenating all UCE loci (Fig. 1). Of 130 internal nodes within Galliformes, only three nodes, all within the Phasianidae, received less than 100% bootstrap support. One was the node between *Chrysolophus* and the clade comprising *Catreus*, *Crossoptilon*, *Lophura*, and *Phasianus* (94%), the second was the node between *Alectoris* and *Perdicula*–*Pternistis* (62%), and the third one was within *Pternistis* (46%). The species tree, which was estimated using SVDquartets [39] on the concatenated sequences from the 75% complete matrix, differed at just three nodes compared to the ML tree (Additional file 1: Fig. S1), but all three of those nodes had low (< 60%) bootstrap support. One of these differences was within Cracidae and the other two were in Phasianidae. Within Cracidae, the ML tree placed *Penelopina* sister to all other guans (*Aburria*, *Chamaepetes*, *Penelope*, and *Pipile*) whereas the species tree united *Penelopina* with *Chamaepetes* (Additional file 1: Fig. S1). For the first of the conflicts in Phasianidae, *Alectoris* was placed as sister to the *Coturnix* clade (*Ammoperdix* and Old World quail) in the species tree (Additional file 1: Fig. S1), rather than sister to the *Perdicula*–*Pternistis* clade in the ML tree (Fig. 1). Finally, the species tree placed *Pternistis bicalcaratus* as sister taxon to the remaining *Pternistis* whereas the ML tree placed *P. ahantensis* in this position (Figs. 1 and Additional file 1: S1). Thus, with a few exceptions, we estimated a broadly sampled and well-supported estimate of galliform relationships.

To test the effects of different gene shopping schemes on divergence time estimation, we selected a group of 48 taxa (including four outgroups and 44 galliform species) to reduce computation time. The ML tree estimated from the 75% complete matrix of the reduced set of 48 taxa was well-supported with only one node receiving less than 100% bootstrap support (97% bootstrap support for the node of *Nothocrax urumutum* and *Mitu salvini* within Cracidae, Additional file 2: Fig. S2). The topologies for the 48 taxa were consistent with those from the 135-taxon dataset.

### Gene shopping schemes for divergence time estimation

**Fig. 2:**
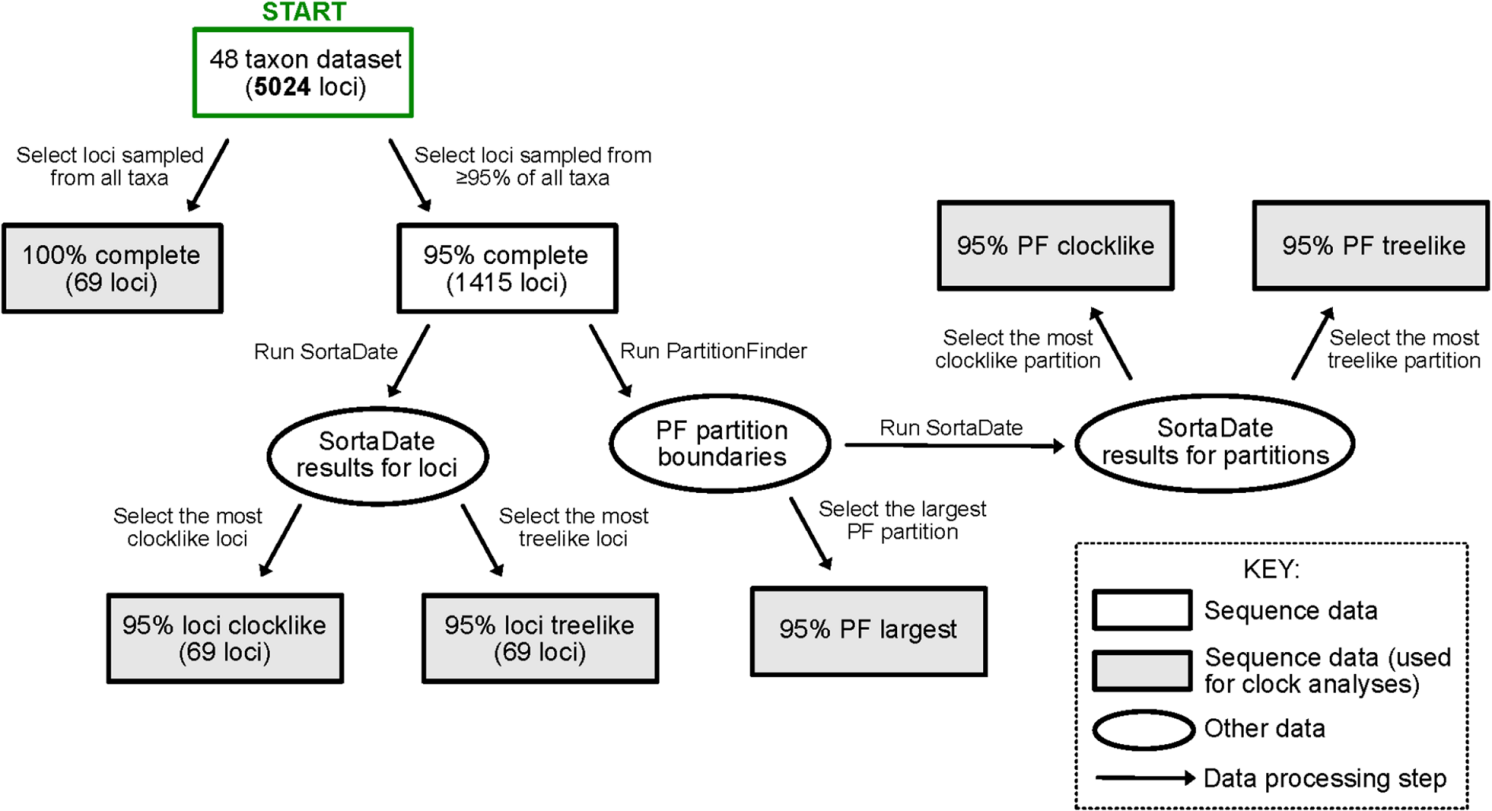
Flow chart of seven different gene shopping schemes for divergence time estimation of the reduced set of 48 taxa. 5,024 UCE locus were aligned from the 48 taxon dataset. To minimize impacts of missing data, we focused on the 100% and 95% complete matrix (greater than 95% of taxa present for each UCE locus). From the 95% complete matrix, we added in two locus-selection schemes, and three partition-selection schemes. For locus selection, we used SortaDate [32] to get 69 (to match the number of loci in the 100% complete matrix) most clock-like (95%-loci-clocklike) and most tree-like (95%-loci-treelike) loci respectively. For partition selection, we used PartitionFinder 32] and SortaDate [40] to get the largest (95%-PF-largest), the most clock-like (95%-PF-clocklike) and the most tree-like (95%-PF-treelike) partition respectively

**Fig. 3:**
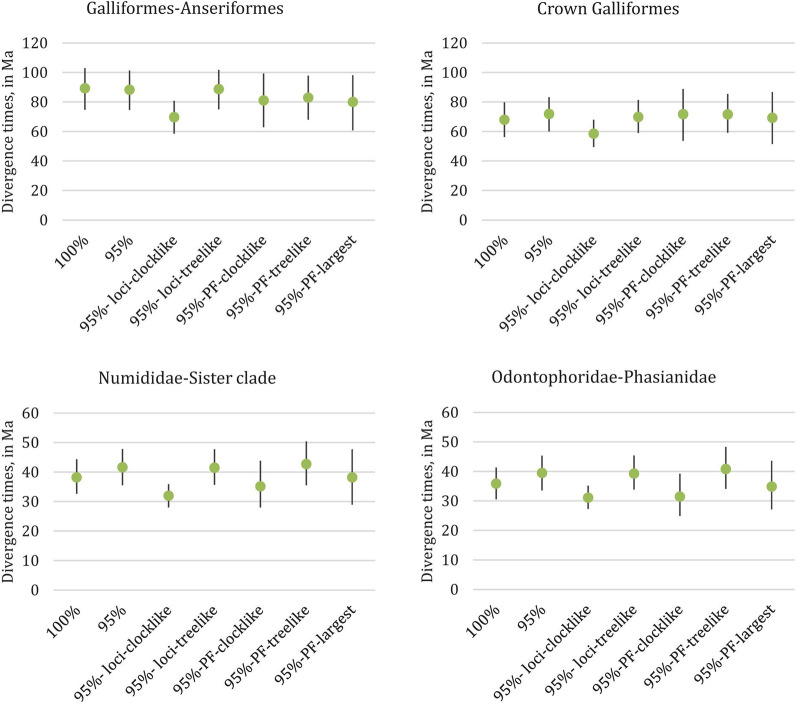
The two clock-like gene shopping schemes and use of the largest partition can result in more recent divergence time estimates for deeper divergences. The point estimate of the divergence time is indicated with a green dot and the bars indicate the 95% HPD. The different gene shopping schemes from left to right are as follows: (1) 100% complete matrix; (2) 95% complete matrix; (3) clocklike loci in 95% complete matrix, (4) treelike loci in 95% complete matrix; (5) most clocklike partition in the 95% complete matrix; (6) most treelike partition in the 95% complete matrix; (7) largest partition in the 95% complete matrix

**Fig. 4:**
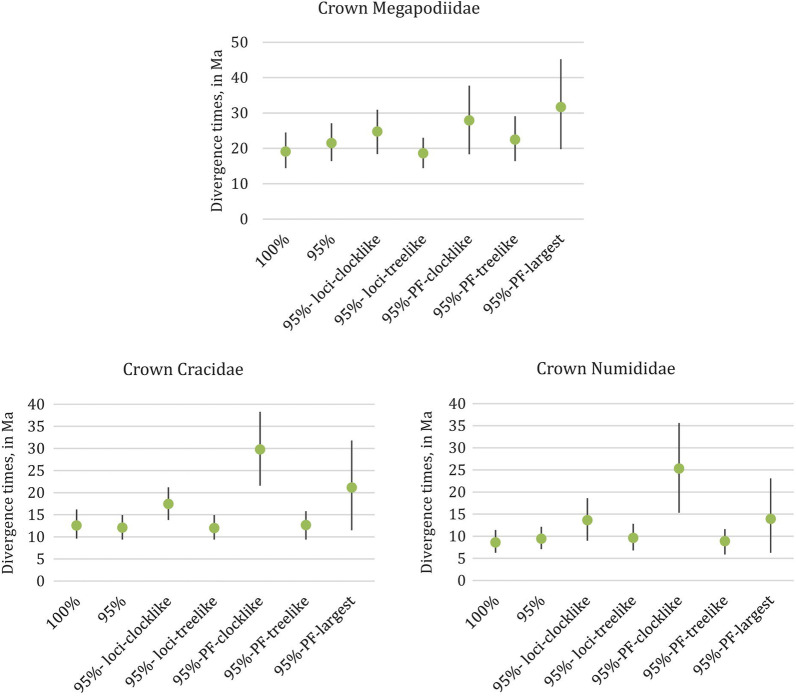
The two clock-like gene shopping schemes and use of the largest partition can result in more ancient divergence time estimates for shallow divergences. The point estimate of the divergence time is indicated with a green dot and the bars indicate the 95% HPD. The different gene shopping schemes from left to right are as follows: (1) 100% complete matrix; (2) 95% complete matrix; (3) clocklike loci in 95% complete matrix, (4) treelike loci in 95% complete matrix; (5) most clocklike partition in the 95% complete matrix; (6) most treelike partition in the 95% complete matrix; (7) largest partition in the 95% complete matrix

We evaluated the impacts of seven different gene shopping schemes that differed in the fraction of loci sampled from the reduced set of 48 taxa. These gene shopping schemes were: (1) the 100% complete matrix (hereafter 100%); (2) the 95% complete matrix (hereafter 95%); (3) the most clocklike loci of the 95% complete matrix (hereafter 95%-loci-clocklike); (4) the most treelike loci (hereafter 95%-loci-treelike); (5) the most clocklike partition from PartitionFinder analysis [40] of the 95% complete matrix (hereafter 95%-PF-clocklike); 6) the most treelike partition (hereafter 95%-PF-treelike); and 7) the largest partition (hereafter 95%-PF-largest) (Fig. 2). There was some variation among all schemes in divergence time estimation (Figs. 3 and 4; Additional file 5: Table S2), though their credible intervals (CIs) partially overlapped in most cases. Only the 95%-PF-clocklike scheme showed a significant difference (*p* < 0.05) to other schemes when comparing all the 49 time points. Restriction to the most clock-like loci or most clock-loci partition sometimes yielded more recent divergence times, particularly for the deeper divergences (Fig. 3, Additional file 5: Table S2). Several nodes also showed increased divergence times in the two clock-like and the largest partition schemes, such as the crown ages of Megapodiidae, Cracidae and Numididae (Fig. 4, Additional file 5: Table S2). Additionally, the two clock-like and the largest partition schemes tended to have wider CIs than the other schemes (Figs. 3 and 4; Additional file 5: Table S2). Increasing the number of loci from 69 to 100 did not substantially change conclusions (Additional file 5: Table S2), suggesting the primary differences were between the gene-shopping scheme, not the number of loci sampled.

We further investigated the potential causes for the variation in divergence times by exploring the characteristics of locus and alignments included in each of the gene shopping schemes, including aligned locus length, GC content, and alpha parameter of the gamma distribution for each locus, and average percent of missing data, average percent of informative sites across each alignment (Table 1). The distribution of locus lengths for each of the gene shopping schemes were very similar (Additional file 3: Fig. S3), only two pairs of the gene shopping scheme (the 95% compared to the 100% and the 95% compared to the 95%-loci-treelike) showed significant differences (*p* < 0.05).We found the alpha parameter and the average percent of informative sites were extremely low in the two clock-like and the largest partition schemes (Table 1). One will obtain low estimates of the alpha parameter when loci have a few sites that change at a very high rate and many sites that change at a very low rate; this is also expected to correlate with a low percentage of informative sites. Moreover, the range of most parameters tended to be narrower in the partition-selection schemes than locus-selection schemes (Table 1). Therefore, in consideration of the variation across different loci within each scheme and the possibly problematic estimates from clock-like schemes, we selected the most tree-like partition from 95% matrix (95%-PF-treelike) as a good scheme for UCE dating (details in discussion).

**Table 1:** Characteristics of the loci included in each gene shopping scheme, and the resulting alignments

	95%	100%	95%-loci-clocklike	95%-loci-treelike	95%-PF-clocklike	95%-PF-treelike	95%-PF-largest
Number of loci	1415	69	69	69	26	54	55
Aligned length among loci	565 (189–1332)	613 (430–1066)	601 (405–1113)	625 (426–1070)	576 (406–765)	555 (337–775)	566 (352–863)
GC content among loci	0.42 (0.30–0.68)	0.47 (0.35–0.64)	0.40 (0.30–0.47)	0.47 (0.34–0.64)	0.37 (0.30–0.41)	0.41 (0.34–0.48)	0.39 (0.35–0.49)
Alpha parameter among loci	0.20 (0.02–29.58)	0.20 (0.02–0.78)	0.02 (0.02–29.58)	0.39 (0.11–1.19)	0.02 (0.02–29.58)	0.34 (0.19–0.79)	0.04 (0.02–12.81)
Length of the alignment	799,310	42,269	41,443	43,109	14,985	30,133	31,041
Average percent of missing data (gaps, ?'s, N's) across alignment	15.70%	14.40%	14.60%	17.20%	15.30%	14.80%	13.70%
Average percent of informative sites across alignment (gaps ignored)	11.40%	14%	2.10%	20.90%	1%	11.90%	2.20%

### Divergence time estimates for Galliformes

**Fig. 5:**
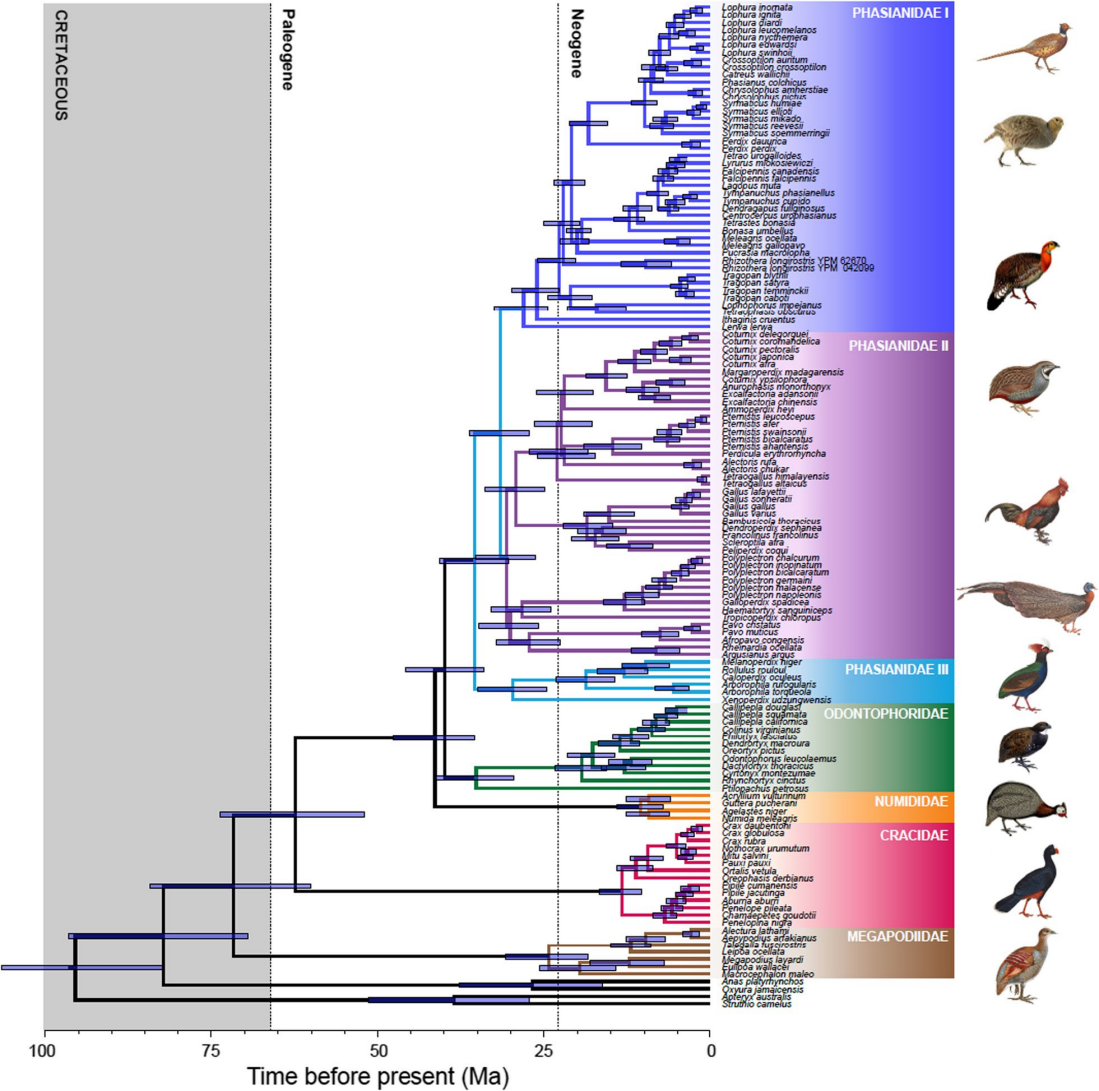
Time tree for Galliformes estimated using the most-tree like partition for the 95% complete 135-taxon dataset. Node bars show the 95% HPD of the divergence times. Family names are indicated using colors and the boxes placed to the right of the tree. Phasianidae, the most species-rich clade in Galliformes, is broken into three subgroups: (I) the “erectile clade” (gallopheasants, true partridges, grouse, turkeys, tragopans, and allies); (II) the “non-erectile clade” (Old world quail, junglefowl, francolins, snowcocks, peafowl, peacock-pheasants, argus pheasants, and allies); and (III) Arborophilinae (Hill partridges and allies). The shaded region is the Cretaceous, and the Paleogene and Neogene are separated by dashed lines

Divergence time estimation for the 135-taxon tree using the most tree-like partition from the 95% complete matrix provided estimates of the timing of divergence for all galliform genera. Under the best gene shopping scheme, the time estimates for the key nodes from the 135 taxa were very similar to those from 48 taxa (Additional file 6: Table S3). The diversification of modern birds (Neornithes) began in the Late Cretaceous (~ 95.5 Ma, Fig. 5), and the divergence between Galliformes and Anseriformes likely occurred before the K-Pg boundary (~ 82.1 Ma, Fig. 5; Table 2). Within Galliformes, Megapodiidae diverged in the Late Cretaceous (~ 71.7 Ma), Cracidae diverged from the remaining families around K-Pg boundary (~ 62.4 Ma), Numididae diverged at ~ 41.3 Ma, and the split between Odontophoridae and Phasianidae occurred at ~ 39.9 Ma (Fig. 5). Divergence times among different genera within each family were relatively recent, mainly during the Neogene (Fig. 5). For example, the crown Megapodiidae, Cracidae, and Numididae began to diversify at ~ 24.3 Ma, ~ 13.3 Ma, ~ 10.6 Ma, respectively.

**Table 2:** Comparison of divergence times (in millions of years, Ma) of major clades in Galliformes between different studies

	This study	Wang et al. 2017 [10]	Stein et al. 2015 [38]	Jetz et al. 2012 [41]	Jarvis et al. 2014 [3]	Prum et al. 2015 with *Vegavis* [5]	Claramunt and Cracraft 2015 [42]	Kuhl et al. 2020 [6]
Anas + Gallus	82.1, 69.5–96.5	74.5, 70.4–79.3	97.8	79.7, 70.5–88.5	65.8, 54.0–74.1	72.4, 68.3–77.6	72.5, 68–79.5	62.5, 57–65
Crown Galliformes	71.7, 60.1–84.2	66.8, 61.9–71.8	95.9, 83.8–108.3	62.0, 47.0–74.1		60.9, 47.8–73.0	58, 47.5–68	55.9, 49–61
Crown Megapodiidae	24.3, 18.4–30.8	19, 15.3–22.8	29.5	38.1, 23.6–53.2				
Cracidae-Sister clade	62.4, 52.0–73.7	61.1, 56.5–66.1	86.4, 75.7–98.7	55.7, 40.7–68.0				
Crown Cracidae	13.3, 10.4–16.7	8.7, 6.7–11.1	21.4, 18.8–24.1	14.6, 9.1–16.7				
Numididae-Sister clade	41.3, 35.4–47.8	44.9, 41.6–48.7	56.8, 49.4–64.5	33.3, 20.6–45.5				
Crown Numididae	10.6, 7.2–14.0	11.8, 9.5–14.5	17.6	15.2, 7.4–22.2				
Odontophoridae-Phasianidae	39.9, 34.0–45.9	42.8, 39.7–46.4	53.6,46.7–61.1	31.1, 17.8–42.1				
Crown Odontophoridae	35.2, 29.5–41.2	39.6, 36.1–43.3	45.2	29.5, 16.8–40.7				
Crown Phasianidae	35.4, 30.3–40.6	36, 33.6–38.9	46.4, 40.6–52.9	25.0, 13.8–35.2	27, 10.5–45			
Core odontophorid	19.4, 15.7–23.3	20.3, 17.3–23.4	27.8	23.4, 11.4–33.9				
Core phasianids	31.5, 27.2–36.2	31.9, 30.1–34.0	42	23.6, 13.2–32.9				

## Discussion

In this study, we estimated a robust phylogenomic tree of Galliformes, that included at least one representative of all extant genera. With only a few exceptions, nodes were well-supported, and the ML tree was mostly congruent with the estimated species tree. Our examination of gene shopping schemes found that some approaches gave very different estimates and identified factors likely to have influenced divergence time estimation. This exploration of the data allowed us to select a suitable gene shopping scheme for divergence time estimation from UCEs. Under this scheme, together with well resolved phylogenetic topology and reliable fossil calibration points, we obtained a robust, well-sampled galliform time tree, which included all 83 extant galliform genera.

### Relationships among the taxa in Galliformes

Our phylogeny was consistent with recent molecular phylogenies in the placement of the major clades [34], though we recovered several relationships within some families that differ from other molecular studies. In Megapodiidae, the mound-building genera (*Leipoa*, *Talegalla*, *Alectura*, and *Aepypodius*) formed a separate clade distinct from the burrow-building genera (*Macrocephalon*, *Megapodius*, and *Eulipoa*) (Fig. 1). While some previous studies have found the burrow-building *Macrocephalon* was united with the mound-builders (albeit with relatively low support [38, 43]), our results are consistent with a recent multilocus study in placing *Macrocephalon* with other burrow-builders [37]. However, our results differ from that study in the order of divergence of the mound-building subclade, which identified *Talegalla* as sister to the remaining three genera [37] rather than *Leipoa* as we identified (Fig. 1).

As noted in the results, relationships within Cracidae conflicted between the ML and species trees within the typical guan lineage (*Aburria*, *Chamaepetes Penelope*, *Penelopina*, and *Pipile*). A recent study analyzed relationships among genera within Cracidae using UCEs (using the same data as in this study), mitochondrial sequences and nuclear introns [11]. Their concatenated and species trees agree with the results of the ML tree in this study (Fig. 1). However, the use of different data types in that study [11] still yield limited support for the weakly supported relationships found in this study. Incomplete lineage sorting and data-type effects (the topological differences associated with the use of different types of markers [44, 45] may have caused the instability within this clade [13, 46].

The relationships among the four genera of Numididae from UCEs exhibited differences from previous studies [38, 47]. A study based on four mitochondrial partitions and one nuclear intron suggested *Guttera* diverged earliest in *Numididae*, with *Agelastes* and *Acryllium* forming a clade [47]. Incorporating more mitochondrial regions and nuclear intron loci [38] found the sister relationship of *Guttera* and *Acryllium* as shown in our UCE phylogeny, but that study lacked the sister relationship we found between *Numida* and *Agelastes*, which instead formed a grade.

Within Odontophoridae, our results agree with the generic relationships obtained using nuclear introns and mitochondrial sequences [48], but with greatly increased bootstrap support.

Phasianidae, the largest galliform family, has received extensive study. Previous studies using relatively few loci exhibited much conflict [34, 35, 49]. Recent UCE studies have helped resolve many of these conflicting relationships [14, 15, 26, 27, 31, 36, 50], largely yielding well-supported relationships such as we found here. We included three newly sequenced genera, *Dendroperdix*, *Peliperdix*, and *Xenoperdix* to provide a comprehensive genus-level topology for the Phasianidae. The placements of these three genera agree with other studies based on limited mitochondrial and nuclear data [10, 38, 47]. There is one clade in the Phasianidae where the ML tree differed from the species tree, albeit with relatively low support in both analyses (Figs. 1 and Additional file 1: Fig. S1). Our previous UCE studies also found the unstable placement of *Alectoris* [26], and even with increased taxon sampling in the Old World quail, the instability still existed [31]. Concatenated ML is thought to have greater power than coalescent methods to identify relationships when incomplete lineage sorting is low, i.e., the relationships among *Alectoris* and *Ammoperdix* [26], though further exploration of the placement of *Alectoris* will need more rapidly evolving markers or improved analytical approaches to provide greater confidence on its position within the family.

### The performance of different gene shopping for divergence time estimation

Although gene shopping has been employed in previous UCE studies [21, 31], such studies have not compared alternative gene shopping schemes on divergence time estimation. However, we found differences among gene shopping schemes on divergence time estimates (Figs. 3 and 4). Missing data is known to have a negative impact on phylogenetic topology estimation [26, 51], and, more importantly, may also bias branch length estimates [52]. Although some studies found missing data had only minor impacts on the accuracy of molecular dating [53], we still decided to use our more complete matrices (100% and 95%) to limit any potential effect of missing data on divergence time estimation. Our seven gene shopping alignments had similar percentages of unresolved nucleotides (Table 1), so any impact of missing data should have affected all datasets equally. The data matrices for the 100%, 95%-loci-clocklike, and 95%-loci-treelike schemes have similar alignment lengths (Table 1). Thus, differences in divergence times (Figs. 3 and 4) cannot be caused by the number of sites in the data matrices. Furthermore, the distributions of locus lengths between different gene shopping schemes are similar (Additional file 3: Fig. S3), and the two clock-like and the largest partition schemes showed no difference to other schemes. Thus, the distribution of locus lengths in each gene shopping scheme cannot explain their differences in divergence time estimation either.

On the other hand, the two clock-like and the largest partition schemes that exhibited the greatest differences in divergence time estimates (extreme values and/or high variance) all had very low percentages of parsimony informative sites (Table 1). Compared to some other molecular markers, UCEs have a very conserved core, with often limited variation in the flanking regions. Thus, it is possible that some UCE loci contain very few informative sites. Loci with few informative sites have little power to reject a molecular clock, so may tend to be selected as more clock-like than other loci. This is likely why the two clock-like schemes (locus and partition) both had a very low percentage of informative sites (Table 1). Similarly, loci with few informative sites likely clustered together into a larger partition, since there would have been little power to identify different patterns of evolution among them. Although the effect of numbers of informative sites on molecular dating has not been tested before, we believe these extremely low variation loci in the two clock-like and the largest partition schemes likely yielded incorrect estimates—they certainly led to estimates that tended to exhibit greater differences from schemes that included more informative loci (Figs. 3 and 4). For phylogenomic studies that primarily include highly informative loci, selecting based on clock-like behavior may be appropriate, but for UCE studies (or other studies) where some loci have little information, care should be taken if clock-like behavior is used to identify loci or partitions for divergence time estimation.

Another common concern for phylogenetic analyses and, more specifically, molecular dating is variation among different loci [29]. Although each locus can be assigned parameters to describe it, this can lead to over-parameterization, which can bias branch length estimates; similarly, describing a set of highly heterogeneous loci with a single set of parameters leads to under-parameterization. Thus, identifying a set of loci that may evolve under similar parameters may be most appropriate for divergence time estimation. Using programs such as PartitionFinder, which cluster loci that exhibit similar parameters allows for selection of partitions that can be used for divergence time estimation [54]. We found the range of most parameters tended to be narrower in the partition-selection schemes than locus-selection schemes (Table 1), implying lower heterogeneity among loci within the alignments from partition-selection schemes. Following this with other criteria, such as SortaDate, may further refine selection of an appropriate partition for divergence time estimation. Thus, we felt the most tree-like partition from our 95% matrix (95%-PF-treelike) would be an appropriate scheme for UCE dating. However, as we noted above, use of the largest or most clock-like partition might be problematic, so care still needs to be taken in selection of which partition may be best.

### Comparison of these UCE divergence time estimates and previous studies

The split between Anseriformes and Galliformes estimated from the 95%-PF-treelike scheme on the 135 taxa dataset is about 82.1 Ma, the CI of this node generally overlapped with recent analyses [3, 5, 10, 41, 42], although with wider CI (Table 2). Increased taxon sampling in the Galliformes resulted in an older crown age than studies of all bird with limited sampling within galliforms (Table 2), except for [41] in which several key nodes were misplaced in the Galliformes [10]. On the other hand, [38] estimated much older ages for these two nodes (Table 2), which might be caused by the absence of distant outgroups (e.g., *Struthio* and *Apteryx* in our study) and improper prior distributions [10], which also lead to older estimations within the Galliformes (Table 2).

The divergence times among the five galliform families were very similar to the ones estimated from mitochondrial and nuclear loci [10]. However, with our increased taxon sampling, well-resolved and strongly-supported topology, and carefully chosen fossil calibrations that are likely to be accurate, the divergence time estimates within each family from UCEs showed several differences between this study and previous studies.

In Megapodiidae, the divergence time estimates from our study were older than those from mitochondrial and nuclear loci [37]. Only one fossil calibration point (at tree root) was used in that study, and the topology within the mound-building subclade was different from our study (see above), which could explain some differences. However, we observed wider CIs in our study than in that study [37], which could be due to the more limited taxon sampling we used (one per genus, rather than all species).

Divergence times within Cracidae from previous study were inferred from mitochondrial and nuclear loci, using the same UCE data in this study as backbone tree, as well as five same fossil calibrations [11]. As expected, their dates for Cracidae were very similar to our UCE dating results, e.g., the origin of crown Cracidae was estimated to be *ca*. 13.1 Ma in that study vs. *ca*. 13.3 Ma in this study, which demonstrated that adequate UCE dating is consistent with dating from mitochondrial and nuclear sequences, and across studies with varying taxonomic representation.

The divergence times for the Numididae and Odontophoridae were also similar between this study and previous multilocus studies [10, 48], which showed the same topologies for sampled taxa, and at least one of the same fossil calibrations was used in their studies.

Overall, our divergence time estimates within Phasianidae were very similar to a recent study [10], e.g., the crown Phasianidae and core phasianids were about 36.0 and 31.9 Ma in that study, while in our analyses they were about 35.4 and 31.5 Ma (Table 2). A few differences were observed, mainly due to added species and altered phylogenetic positions. For example, the inclusion of *Lerwa* pushed the time to the most recent common ancestor (TMRCA) of Erectile clade 2 Ma earlier, from 26.1 to 28.1 Ma; the inclusion of *Tropicoperdix* also pushed the TMRCA of *Polyplectron* clade significantly earlier (Fig. 5). Previous multilocus studies [10] have found *Pavo* and *Polyplectron* to form grade, while we found strong support for sister relationship (Fig. 1), as in other UCE studies [14], changing the basal divergences within Non-erectile Clade (Fig. 5). The deep divergence of our two *Rhizothera* samples (one from Myanmar and one from Borneo) is a potentially surprising result; the same two accessions also exhibit a high degree of mitochondrial divergence [27], suggesting that they may represent good candidates for taxa that should be split into distinct species.

## Conclusions

We generated a well-resolved galliform phylogeny that broadly sampled all genera, to allow robust estimates of divergence times, providing insights into the evolution of extant galliforms. Such phylogenies are important, as reconstruction of traits or ancestral ranges can be highly biased by phylogenies that are inaccurate or have biased taxon sampling [14, 16, 31, 55], and interpretation of such results will be in error if the timing of the events has been mis-estimated. Furthermore, our well resolved galliform tree now provides a robust backbone time tree that can be combined with more fossil records to further facilitate our understanding for the evolution of Galliformes [56] and as a resource for comparative and biogeographic studies of this interesting group [57–59].

## Materials and methods

### DNA sequencing and data processing

We sequenced one species from each galliform genus not represented in previous studies (Additional file 4: Table S1). To do this, we extracted genomic DNA from tissues using the tissue protocol for the PUREGENE® DNA Purification Kit (Qiagen). UCEs were sequenced by RAPiD Genomics (Gainesville, FL) using protocols modified from BC Faircloth, JE McCormack, NG Crawford, MG Harvey, RT Brumfield and TC Glenn [18]. Briefly, Illumina TruSeq libraries were prepared using the manufacturer’s protocol (Illumina Inc., San Diego, CA, USA) modified to use primers with custom index tags [60]. Each library was enriched for 5060 UCE loci targeted using a set of 5472 probes (Mycroarray, Ann Arbor, MI; http://www.mycroarray.com/mybaits/mybaits-UCEs.html) and 100nt paired-end reads were generated using an Illumina (San Diego, CA) HiSeq 2500. We removed PCR duplicates from demultiplexed reads with PrinSeq-lite 0.20.4 [61], poor-quality reads were cleaned and adapter reads were trimmed using Trimmomatic 0.36 [62]. We then assembled quality-controlled reads into contigs with Trinity r20150302 [63].

We added these newly sequenced species to data from previous studies (Additional file 4: Table S1, [11, 14, 26, 27, 31, 36, 50] to obtain UCEs from 130 galliform species, including representatives of all genera, except the genus *Ophrysia*, which is thought to have gone extinct in the 1800’s. We also included UCEs from the ruddy duck (*Oxyura jamaicensis*) [26] and harvested UCEs from published genome data for the ostrich (*Struthio camelus*), kiwi (*Apteryx australis*), and mallard (*Anas platyrhynchos*) from GenBank (GCA_000698965.1, GCA_001039765.2, and GCA_000355885.1 respectively) to provide more distant outgroups to reduce the stochastic error in time estimation [10], resulting in 135 taxa. UCE sequences from those three published genomes were extracted using PHYLUCE as described in the PHYLUCE documentation https://phyluce.readthedocs.io/en/latest/index.html. We produced our data matrices using the standard PHYLUCE [64] pipeline: first we extracted UCEs from contigs, then we aligned each UCE locus using MAFFT 7 [65] using the standard settings for the PHYLUCE pipeline, edge-trimmed the alignments, and finally generate concatenated alignments that included UCE loci sampled for 100%, > 95%, and > 75% of taxa.

To test the effects of different gene shopping schemes on divergence time estimation (see below), we used a subset of the taxa to reduce computation time, using a data matrix generated using PHYLUCE as described above. For these analyses, we selected 44 galliform species including all genera from Megapodiidae, Cracidae, Numididae, Odontophoridae, and eight genera from Phasianidae, representing all the three major clades identified within Phasianidae [34], as well as all four outgroups to form a reduced group of 48 taxa. As much as possible, we focused on taxa with higher quality data (more UCEs recovered).

### Phylogenetic analysis

We used RAxML 8.2.12 [66] under a best tree plus 100 rapid bootstrap replicates (‘-f a’ option) using GTR + G model to estimate the ML trees for the 75% complete matrix of both the 135-taxon and 48-taxon datasets. The 75% criterion was selected based on the analyses of galliform UCEs [26]. For the 135-taxon dataset, we estimated a species tree using SVDquartets [39] implemented in PAUP*4.0a168 [67]; 100 bootstrap searches were performed and all possible quartets were evaluated in each search.

For the 48-taxon dataset, the ML tree from the 75% complete matrix was used as guide tree for divergence time estimation. However, we used the sequence data in the 95% and 100% complete matrix for divergence time estimation (see below).

### “Gene shopping” schemes for divergence time estimation

We conducted divergence time estimation using a total of seven schemes based on the 48-taxon dataset (Fig. 2). To minimize impacts of missing data, which can bias branch length estimation [23, 52], we focused on the 95% and 100% complete matrix. This included the 95% matrix (95%; 1415 loci) and the 100% complete matrix (100%; 69 loci). Besides, we added in two locus-selection schemes, and three partition-selection schemes. For locus selection, we used SortaDate [32], which uses three criteria to select loci (clock-like, tree-like, and tree length); the order these three criteria are applied can be selected to yield datasets focused on different loci. To allow for a good comparison that would be independent of numbers of loci analyzed, we selected 69 loci (to match the number of loci in the 100% matrix) from the 1415 loci in the 95% complete matrix. To get the most clock-like loci, we set SortaDate to use clock-like first, followed by tree-like and then tree length (95%-loci-clocklike); we did a second selection to obtain the 69 most tree-like loci by considering tree-like as the primary criterion, followed by clock-like then tree length (95%-loci-treelike) loci from the 95% complete matrix. Tree-like identifies loci that mostly closely match a species tree, and thus should select a set of loci with a coalescent time similar to the speciation time. We did not prioritize tree length, as it is suggested to identify loci with high information content [32]. High information content loci should also be selected using the tree-like criterion (since too little information would not have the power to estimate a tree similar to the species tree, so using tree-like should yield the benefits of tree length and tree-like criteria together).

To avoid over-fitting models, phylogenetic analyses on large datasets frequently combine loci into larger partitions [68]. Methods that identify appropriate partitions, such as PartitionFinder [54] do so by grouping loci with similar evolutionary parameters. Thus, a partition should have limited locus heterogeneity, and may be more appropriate for divergence time estimation. For partition-based gene shopping schemes, we first selected the optimal partitioning scheme on the 95% complete matrix using the Bayesian information criterion (BIC) and the rclusterf algorithm [54] in PartitionFinder 2 [40], branch lengths were set to linked and GTR + G model was used. Then we used SortaDate [32] on the resulting 58 partitions to select the most clock-like (95%-PF-clocklike) and most tree-like (95%-PF-treelike) partition respectively. We also conducted divergence time estimation on the largest partition from PartitionFinder (95%-PF-largest). We could not control the number of loci in the selected partition (unlike with locus selection, where we selected 69 loci that best fit each criterion), so the numbers of loci included in these analyses were variable. The divergence times for each of the gene shopping schemes (49 time points for each scheme) were compared using the Independent-Samples Kruskal–Wallis Test in SPSS 26.

We investigated the parameters of our different schemes. For each locus included in one of the schemes, we obtained the aligned locus length, the GC content, and alpha parameter of the gamma distribution from RAxML analyses. We compared the locus lengths for each of the gene shopping schemes using the Independent-Samples Kruskal–Wallis Test in SPSS 26. We also used the python script Alignment Assessment [69] to estimate percentage of data represented by? and N (this is a combination of loci not sampled for an included species, which should be relatively limited since we focused on more complete matrices, but also cases where assembled contigs were much shorter in some species leaving many unresolved nucleotides), and percentage of informative sites across the seven alignments.

### Divergence time estimation

For all the seven alignments from the 48-taxon dataset, we implemented MCMCTREE (PAML 4.9j [70]) with approximate likelihood calculation, using the topology estimated from the 75% complete matrix. A number of early studies focused on galliform divergence times have used problematic fossils for calibration [10, 71]. For example, the inappropriate phylogenetic position of *Gallinuloides wyomingensis* has led to overestimation of divergence times in Galliformes [71]. Therefore, we used six carefully chosen galliform fossils that were validated by previous studies as lower minimal bounds for node ages (Additional file 7: Table S4) [10, 11, 31]. We set a maximum age constraint of 99.6 million years ago (Ma) for the tree root (the most recent common ancestor of Neornithes) based on the Early-Late Cretaceous boundary (also used in other avian phylogenomic studies [3, 72]). The parameter settings of MCMCTREE were as follows: clock = 2, RootAge ≤ 9.96, model = 4, BDparas = 1 1 0, kappa_gamma = 6 2, alpha_gamma = 1 1, sigma2_gamma = 1 4.5, rgene_gamma was determined by the substitution rate estimated from BASEML [73]. We ran all analyses twice to assess convergence, the effective sample sizes of all parameters were checked in Tracer 1.7.1 [74] to ensure they were above 200.

Using what our results suggested was an appropriate gene shopping scheme (95%-PF-treelike, see above), we estimated divergence times of Galliformes on the ML topology from the 135-taxon dataset using the same methods described above.

## Supplementary Information

**Additional file 1: Figure S1.** Species tree for the Galliformes from the 135-taxon dataset. Estimation was conducted on the 75% complete matrix using SVDquartets.

**Additional file 2: Figure S2.** ML phylogeny for the Galliformes from the 48-taxon dataset. Estimation was conducted on the 75% complete matrix using RaxML.

**Additional file 3: Figure S3.** Boxplot for the locus lengths for each of the gene shopping schemes.

**Additional file 4: Table S1.** UCE phylogenomic studies of Galliformes and novel UCEs used in this study.

**Additional file 5: Table S2.** Divergence time estimations (in 10 millions of years) of different gene shopping schemes for the 48-taxon dataset.

**Additional file 6: Table S3.** Divergence time estimations (in 10 millions of years) from the most-tree like partition for the 95% complete of the 48 and 135-taxon datasets.

**Additional file 7: Table S4.** Fossils used to time-calibrated the galliform phylogeny.

## Data Availability

Sequence reads of the newly sequenced species in this study has been deposited to GenBank: PRJNA733296. All sequence alignments and tree files are available at Mendeley Data: https://doi.org/10.17632/gcwxcmysw7.2 (https://data.mendeley.com/datasets/gcwxcmysw7/1).

## Abbreviations

CI: Credible interval
Ma: Million years ago
ML: Maximum likelihood
UCE: Ultraconserved element
