# Supplementary figures and images for: Divergence time estimation of Galliformes based on the best gene shopping scheme of ultraconserved elements
Source: BMC Ecol Evol. 2021 Nov 22;21:209. doi: 10.1186/s12862-021-01935-1 (PMC8609756; doi:10.1186/s12862-021-01935-1)

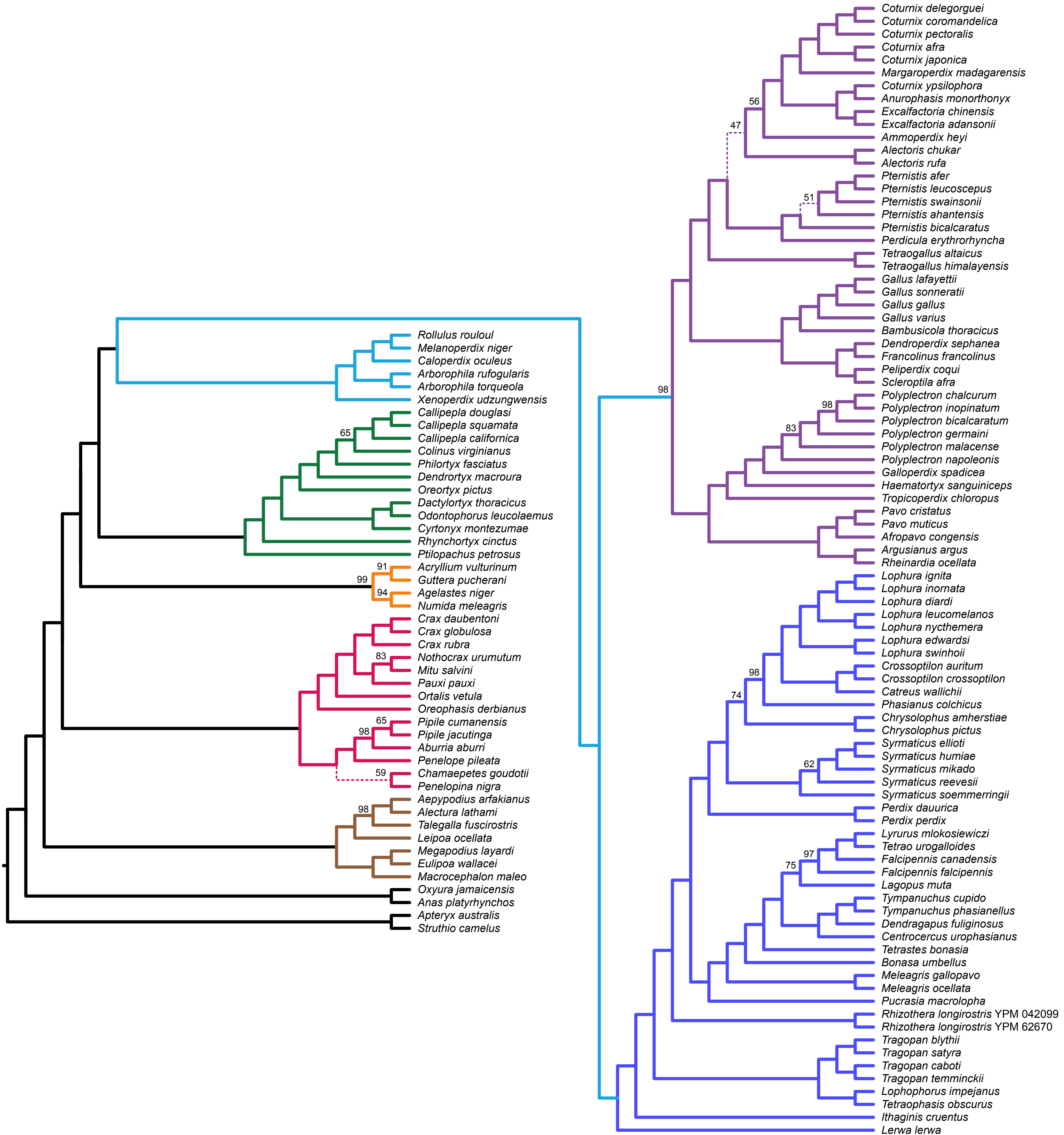

Supplement: Supplementary file 1 — Additional file 1: Figure S1. Species tree for the Galliformes from the 135-taxon dataset. Estimation was conducted on the 75% complete matrix using SVDquartets. [file 12862_2021_1935_MOESM1_ESM.pdf]

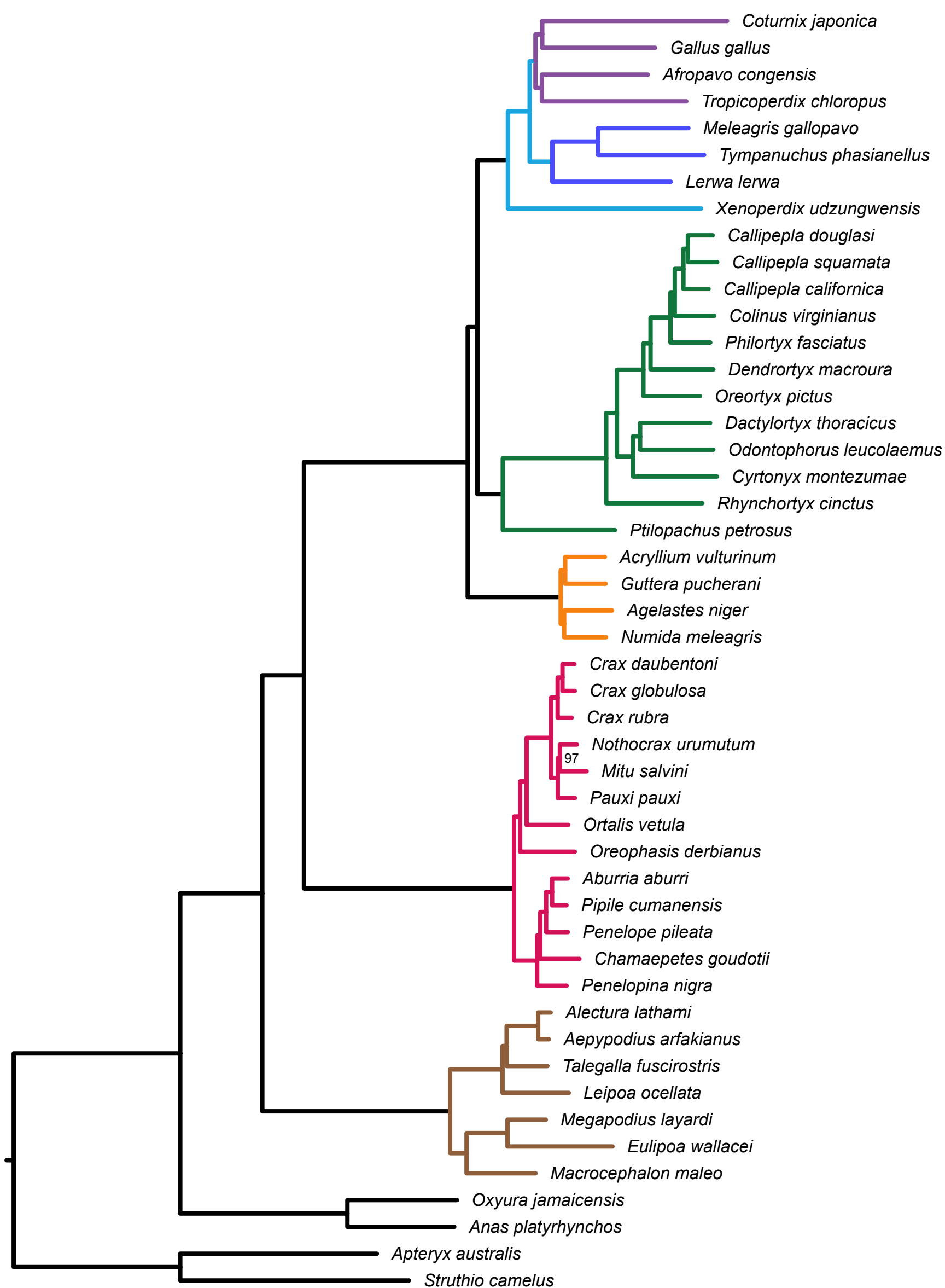

0.0080

Supplement: Supplementary file 2 — Additional file 2: Figure S2. ML phylogeny for the Galliformes from the 48-taxon dataset. Estimation was conducted on the 75% complete matrix using RaxML. [file 12862_2021_1935_MOESM2_ESM.pdf]

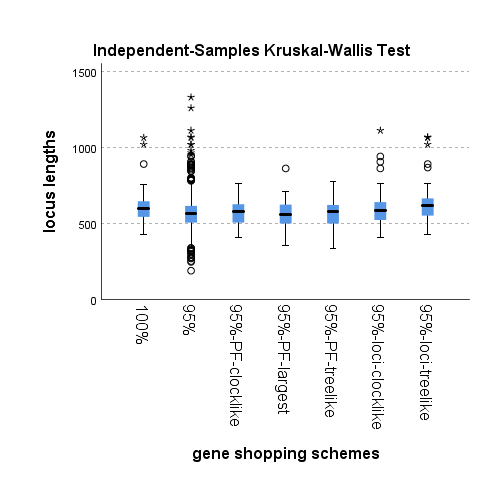

Supplement: Supplementary file 3 — Additional file 3: Figure S3. Boxplot for the locus lengths for each of the gene shopping schemes. [file 12862_2021_1935_MOESM3_ESM.docx]
