# Supplementary material for: Divergence time estimation of Galliformes based on the best gene shopping scheme of ultraconserved elements
Source: BMC Ecol Evol. 2021 Nov 22;21:209. doi: 10.1186/s12862-021-01935-1 (PMC8609756; doi:10.1186/s12862-021-01935-1)
Supplement: Supplementary file 5 — Additional file 5: Table S2. Divergence time estimations (in 10 millions of years) of different gene shopping schemes for the 48-taxon dataset. [file 12862_2021_1935_MOESM5_ESM.docx]

Table S2. Divergence time estimations (in 10 millions of years) of different gene shopping schemes for the 48-taxon dataset.

|  | 100% | 95% | 95%- loci-clocklike (69 loci) | 95%- loci-treelike (69 loci) | 95%-PF-clocklike | 95%-PF-treelike | 95%-PF-largest | 95%- loci-clocklike (100 loci) | 95%- loci-treelike (100 loci) |
| --- | --- | --- | --- | --- | --- | --- | --- | --- | --- |
| Galliformes-Anseriformes | 8.93, 7.47-10.3 | 8.83, 7.45-10.13 | 6.98, 5.86-8.08 | 8.88, 7.5-10.18 | 8.11, 6.3-9.93 | 8.29, 6.81-9.79 | 8.01, 6.08-9.82 | 7.76, 6.25-9.45 | 8.84, 7.48-10.09 |
| Crown Galliformes | 6.81, 5.63-7.98 | 7.2, 6.01-8.33 | 5.86, 4.95-6.81 | 6.99, 5.9-8.14 | 7.18, 5.37-8.89 | 7.17, 5.92-8.55 | 6.93, 5.15-8.68 | 6.32, 5.01-7.71 | 7.04, 5.98-8.15 |
| Crown Megapodiidae | 1.91, 1.44-2.45 | 2.15, 1.64-2.71 | 2.48, 1.84-3.09 | 1.86, 1.44-2.3 | 2.79, 1.83-3.77 | 2.25, 1.64-2.91 | 3.17, 1.98-4.52 | 2.56, 1.81-3.34 | 1.93, 1.53-2.41 |
| Cracidae-Sister clade | 5.89, 4.85-6.99 | 6.35, 5.27-7.38 | 5.32, 4.44-6.19 | 6.13, 5.16-7.19 | 6.26, 4.63-7.96 | 6.22, 5.06-7.42 | 5.89, 4.27-7.56 | 5.75, 4.55-7.08 | 6.2, 5.22-7.21 |
| Crown Cracidae | 1.26, 0.96-1.62 | 1.21, 0.94-1.49 | 1.75, 1.38-2.12 | 1.2, 0.94-1.49 | 2.98, 2.16-3.83 | 1.27, 0.94-1.58 | 2.12, 1.15-3.18 | 1.55, 1.11-2.01 | 1.18, 0.93-1.45 |
| Numididae-Sister clade | 3.82, 3.26-4.43 | 4.16, 3.55-4.78 | 3.2, 2.8-3.59 | 4.15, 3.57-4.77 | 3.52, 2.8-4.38 | 4.27, 3.55-5.04 | 3.82, 2.89-4.77 | 3.67, 3.02-4.42 | 4.16, 3.57-4.73 |
| Crown Numididae | 0.86, 0.63-1.14 | 0.94, 0.71-1.21 | 1.36, 0.9-1.86 | 0.96, 0.68-1.28 | 2.53, 1.53-3.56 | 0.89, 0.59-1.18 | 1.39, 0.63-2.31 | 0.99, 0.66-1.33 | 0.92, 0.69-1.19 |
| Odontophoridae-Phasianidae | 3.59, 3.05-4.13 | 3.95, 3.36-4.53 | 3.11, 2.73-3.52 | 3.93, 3.39-4.54 | 3.15, 2.49-3.92 | 4.08, 3.41-4.83 | 3.49, 2.71-4.36 | 3.48, 2.85-4.17 | 3.95, 3.4-4.51 |
| Crown Odontophoridae | 3.17, 2.64-3.71 | 3.43, 2.89-4 | 2.71, 2.3-3.16 | 3.45, 2.94-4.04 | 2.81, 2.12-3.59 | 3.48, 2.81-4.15 | 3.1, 2.26-4.04 | 2.86, 2.22-3.53 | 3.46, 2.93-4.01 |
| Crown Phasianidae | 3.15, 2.7-3.62 | 3.4, 2.94-3.92 | 3.03, 2.65-3.44 | 3.41, 2.96-3.93 | 3.05, 2.39-3.77 | 3.62, 3.01-4.27 | 3.3, 2.57-4.12 | 3.22, 2.66-3.83 | 3.43, 2.96-3.92 |
| Core odontophorid | 1.94, 1.54-2.37 | 2.05, 1.66-2.45 | 1.78, 1.44-2.12 | 2.09, 1.72-2.51 | 2.46, 1.7-3.24 | 2.07, 1.61-2.56 | 2.02, 1.15-2.87 | 1.63, 1.19-2.05 | 2.07, 1.7-2.49 |
| Core phasianids | 2.84, 2.45-3.25 | 3, 2.6-3.45 | 2.88, 2.51-3.28 | 3.02, 2.63-3.46 | 2.95, 2.31-3.66 | 3.16, 2.65-3.72 | 3, 2.35-3.73 | 2.93, 2.43-3.46 | 3, 2.59-3.39 |
