# Supplementary material for: Divergence time estimation of Galliformes based on the best gene shopping scheme of ultraconserved elements
Source: BMC Ecol Evol. 2021 Nov 22;21:209. doi: 10.1186/s12862-021-01935-1 (PMC8609756; doi:10.1186/s12862-021-01935-1)
Supplement: Supplementary file 6 — Additional file 6: Table S3. Divergence time estimations (in 10 millions of years) from the most-tree like partition for the 95% complete of the 48 and 135-taxon datasets. [file 12862_2021_1935_MOESM6_ESM.docx]

|  | 95%-PF-treelike (48 taxon) | 95%-PF-treelike (135 taxon) |
| --- | --- | --- |
| Galliformes-Anseriformes | 8.29, 6.81-9.79 | 8.21, 6.95-9.65 |
| Crown Galliformes | 7.17, 5.92-8.55 | 7.17, 6.01-8.42 |
| Crown Megapodiidae | 2.25, 1.64-2.91 | 2.43, 1.84-3.08 |
| Cracidae-Sister clade | 6.22, 5.06-7.42 | 6.24, 5.2-7.37 |
| Crown Cracidae | 1.27, 0.94-1.58 | 1.33, 1.04-1.67 |
| Numididae-Sister clade | 4.27, 3.55-5.04 | 4.13, 3.54-4.78 |
| Crown Numididae | 0.89, 0.59-1.18 | 1.06, 0.72-1.4 |
| Odontophoridae-Phasianidae | 4.08, 3.41-4.83 | 3.99, 3.4-4.59 |
| Crown Odontophoridae | 3.48, 2.81-4.15 | 3.52, 2.95-4.12 |
| Crown Phasianidae | 3.62, 3.01-4.27 | 3.54, 3.03-4.06 |
| Core odontophorid | 2.07, 1.61-2.56 | 1.93, 1.57-2.33 |
| Core phasianids | 3.16, 2.65-3.72 | 3.15, 2.72-3.62 |

Table S3. Divergence time estimations (in 10 millions of years) from the most-tree like partition for the 95% complete of the 48 and 135-taxon datasets.
