# Supplementary material for: Divergence time estimation of Galliformes based on the best gene shopping scheme of ultraconserved elements
Source: BMC Ecol Evol. 2021 Nov 22;21:209. doi: 10.1186/s12862-021-01935-1 (PMC8609756; doi:10.1186/s12862-021-01935-1)
Supplement: Supplementary file 7 — Additional file 7: Table S4. Fossils used to time-calibrated the galliform phylogeny. [file 12862_2021_1935_MOESM7_ESM.docx]

| Taxon | locality | node calibrated | hard minmum |
| --- | --- | --- | --- |
| *Gallinuloides wyomingensis* | Green River, Wyoming, USA | Galliformes + Anseriformes | 51.6 Ma |
| *Palaeortyx gallica* | Enspel, Germany | basal Phasianidae | 24.5 Ma |
| *Schaubortyx keltica* | Armissan, France | Odontophoridae + Phasianidae | 27.5 Ma |
| *Rhegminornis calobates* | Gilchrist Co., Florida, USA | *Meleagris* + *Tympanuchus* | 18.0 Ma |
| *Progura gallinacea* | Darling Downs, Australia | *Leipoa* + *Alectura* | 4.5 Ma |
| *Callipepla shotwelli* | Umatilla Co., Oregon, USA | *Callipepla* + *Colinus* | 3.5 Ma |

Table S3. Fossils used to time-calibrated the galliform phylogeny.
